# Supplementary figures and images for: The rsmA mutant from Pseudomonas aeruginosa ID4365 is a non-virulent strain that is suitable for pyocyanin and phenazine-1-carboxylic acid production
Source: PLoS One. 2025 Dec 4;20(12):e0337097. doi: 10.1371/journal.pone.0337097 (PMC12677446; doi:10.1371/journal.pone.0337097)

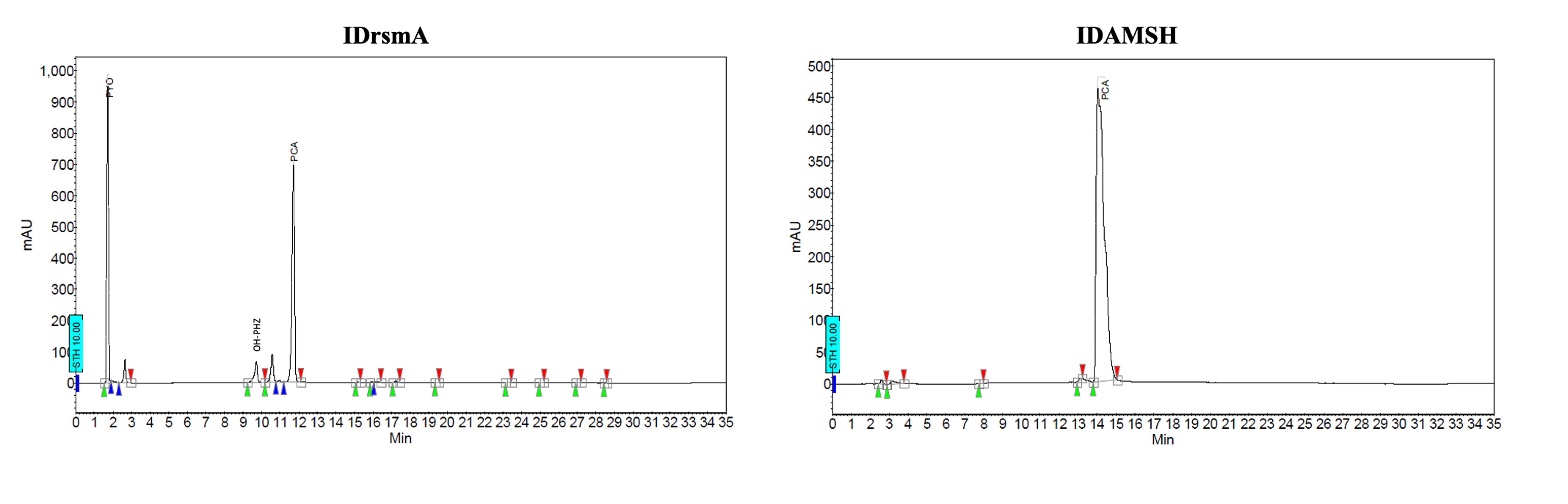

Supplement: S3 Fig — At 24 hours, its chromatogram shows only PCA, while the IDrsmA strain produces pyocyanin (PYO), PCA and OH-PHZ. (TIFF) [file pone.0337097.s003.tiff]
