# Supplementary material for: The rsmA mutant from Pseudomonas aeruginosa ID4365 is a non-virulent strain that is suitable for pyocyanin and phenazine-1-carboxylic acid production
Source: PLoS One. 2025 Dec 4;20(12):e0337097. doi: 10.1371/journal.pone.0337097 (PMC12677446; doi:10.1371/journal.pone.0337097)
Supplement: S1 Table — (DOCX) [file pone.0337097.s004.docx]

Table S1. Bacterial strains used in this work.

| ***P. aeruginosa* strains** | | |
| --- | --- | --- |
| **Strain** | **Description** | **Reference** |
| UCBPP-PA14 | Human burn patient | [1] |
| ATCC9027 | Outer-ear isolate | ATCC collection. |
| ID4365 | Wild type strain isolated from Indic Ocean. | [2] |
| IDrsmA | ID4365 *rsmA* deletion mutant, Tc^R^ | [3] |
| IDrsmA/pUCrsmA | IDrsmA strain harbouring plasmid pUCrsmA, Tc^R^ Cb^R^ | [3] |
| IDrsmA/pUCP20 | IDrsmA strain harbouring plasmid pUCP20, Tc^R^ Cb^R^ | [3] |
| IDMSH | ID4365 derivative strain carrying deletions in *phzM*, *phzS* and *phzH*. | This work |
| IDAMSH | IDrsmA derivative strain carrying deletions in *phzM*, *phzS* and *phzH*. Tc^R^ | This work |
| *E. coli* strains | | |
| *Escherichia coli* DH5α | φ80Δ*lacZ*ΔM15Δ[*lacZYA*-*argF*]U169 *endA recA1 hsdR17* deoRthi-1supE44 | Invitrogen |

References

1. Lee DG, Urbach JM, Wu G, Liberati NT, Feinbaum RL, Miyata S, et al. Genomic analysis reveals that *Pseudomonas aeruginosa* virulence is combinatorial. Genome Biol. 2006;7: R90. doi:10.1186/gb-2006-7-10-r90

2. Manwar AV, Khandelwal SR, Chaudhari BL, Meyer JM, Chincholkar SB. Siderophore production by a marine *Pseudomonas aeruginosa* and its antagonistic action against phytopathogenic fungi. ABAB. 2004;118: 243–252. doi:10.1385/ABAB:118:1-3:243

3. Cocotl-Yañez M, Soto-Aceves MP, González-Valdez A, Servín-González L, Soberón-Chávez G. Virulence factors regulation by the quorum-sensing and Rsm systems in the marine strain *Pseudomonas aeruginosa* ID4365, a natural mutant in *lasR*. FEMS Microbiol Lett. 2020;367: fnaa092. doi:10.1093/femsle/fnaa092
