# Supplementary material for: The rsmA mutant from Pseudomonas aeruginosa ID4365 is a non-virulent strain that is suitable for pyocyanin and phenazine-1-carboxylic acid production
Source: PLoS One. 2025 Dec 4;20(12):e0337097. doi: 10.1371/journal.pone.0337097 (PMC12677446; doi:10.1371/journal.pone.0337097)
Supplement: S2 Table — (DOCX) [file pone.0337097.s005.docx]

Table S2. Plasmids used in this work.

| **Plasmid** | **Description** | **Reference** |
| --- | --- | --- |
| pJET1.2/blunt | Cloning vector | Thermo Scientific |
| pJetM | pJET1.2/blunt derived plasmid containing the deletion allele to replace the *phzM* gene with apramycin cassette flanked with HindIII restriction site. | This work |
| pJetS | pJET1.2/blunt derived plasmid containing the deletion allele to replace the *phzS* gene with apramycin cassette flanked with EcoRI restriction site. | This work |
| pJetH | pJET1.2/blunt derived plasmid containing the deletion allele to replace the *phzH* gene with apramycin cassette flanked with HindIII restriction site. | This work |
| pEX18:Sm | Gene replacement vector, Sm^R^. | [1] |
| pEX18:Amp | Gene replacement vector, Amp^R^ | [2] |
| pEXM | pEX18:Amp derived plasmid containing the deletion allele to replace the *phzM* gene with apramycin cassette. | This work |
| pEXS | pEX18:Sm derived plasmid containing the deletion allele to replace the *phzS* gene with apramycin cassette. | This work |
| pEXH | pEX18:Amp derived plasmid containing the deletion allele to replace the *phzH* gene with apramycin cassette. | This work |
| pFLP2 | Plasmid vector expressing FLP-recombinase. Amp^R^ | [2] |
| pIJ773 | Plasmid carrying the apramycin gene resistance. Apra^R^ | [3] |
|  |  |  |

Antibiotics resistance abbreviations: Tc^R^ (tetracycline), Sm^R^ (streptomycin), Apra^R^ (apramycin), Cb^R^ (carbenicillin), Amp^R^ (ampicillin).

References

1. García‐Reyes S, Cocotl‐Yañez M, Soto‐Aceves MP, González‐Valdez A, Servín‐González L, Soberón‐Chávez G. PqsR‐independent quorum‐sensing response of *Pseudomonas aeruginosa* ATCC 9027 outlier‐strain reveals new insights on the PqsE effect on RhlR activity. Mol Microbiol. 2021;116: 1113–1123. doi:10.1111/mmi.14797

2. Hoang TT, Karkhoff-Schweizer RR, Kutchma AJ, Schweizer HP. A broad-host-range Flp-FRT recombination system for site-specific excision of chromosomally-located DNA sequences: application for isolation of unmarked *Pseudomonas aeruginosa* mutants. Gene. 1998;212: 77–86. doi:10.1016/S0378-1119(98)00130-9

3. Gust B, Challis GL, Fowler K, Kieser T, Chater KF. PCR-targeted *Streptomyces* gene replacement identifies a protein domain needed for biosynthesis of the sesquiterpene soil odor geosmin. Proc Natl Acad Sci USA. 2003;100: 1541–1546. doi:10.1073/pnas.0337542100
