# Supplementary material for: The rsmA mutant from Pseudomonas aeruginosa ID4365 is a non-virulent strain that is suitable for pyocyanin and phenazine-1-carboxylic acid production
Source: PLoS One. 2025 Dec 4;20(12):e0337097. doi: 10.1371/journal.pone.0337097 (PMC12677446; doi:10.1371/journal.pone.0337097)
Supplement: S3 Table — (DOCX) [file pone.0337097.s006.docx]

Table S3. Oligonucleotides used in this study.

|  | **Name** | **Oligonucleotide sequence 5’ – 3’** |
| --- | --- | --- |
| *phzH* mutant | 5’phzHUp | TGTAAGCTTAACGTTGCCACGAAATCAG |
|  | 3’phzHUp | GTCGACGGATCCCCGGAATAGCACATAGGGAAACTCCTCT |
|  | 5’phzHDw | GAAGCAGCTCCAGCCTACATTGTACGGGCTCTCCGCCTGA |
|  | 3’phzHDw | CTTAAGCTTACAAGCGCAGGACGCCCGAT |
| *phzM* mutant | 5’phzMUp | TGTAAGCTTTCATCCCGGGTTTCTTTTGA |
|  | 3’phzMUp | GTCGACGGATCCCCGGAATACTCTCTCGTTACACATTTCC |
|  | 5’phzMDw | GAAGCAGCTCCAGCCTACAATCGGAACTCTCAACGGTTG |
|  | 3’phzMDw | CTTAAGCTTTTTCTTCGCCCTGCGCAGC |
| *phzS* mutant | 5’phzSUp | TGGGAATTCACCGCTACCTGCAACCGTGA |
|  | 3’phzSUp | GTCGACGGATCCCCGGAATACTTTCCAGCAGCGTGACCTT |
|  | 5’phzSDw | GAAGCAGCTCCAGCCTACAACGCTAGCAACACCGGGCA |
|  | 3’phzSDw | CTTGAATTCTGTGGCGCGGTTTCGCCCTT |
