# Supplementary material for: The rsmA mutant from Pseudomonas aeruginosa ID4365 is a non-virulent strain that is suitable for pyocyanin and phenazine-1-carboxylic acid production
Source: PLoS One. 2025 Dec 4;20(12):e0337097. doi: 10.1371/journal.pone.0337097 (PMC12677446; doi:10.1371/journal.pone.0337097)

Supernatant (Fig. 1)

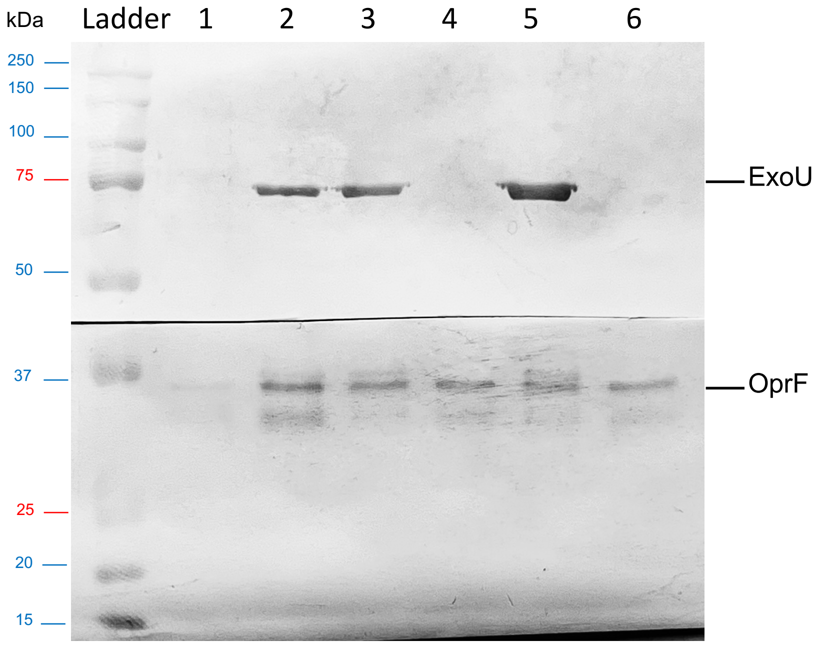

Well	Name
1	ATCC9027
2	PA14
3	ID4365
4	IDrsmA
5	IDrsmA + pUCrsmA
6	IDrsmA + pUCP20

Biological replicates:

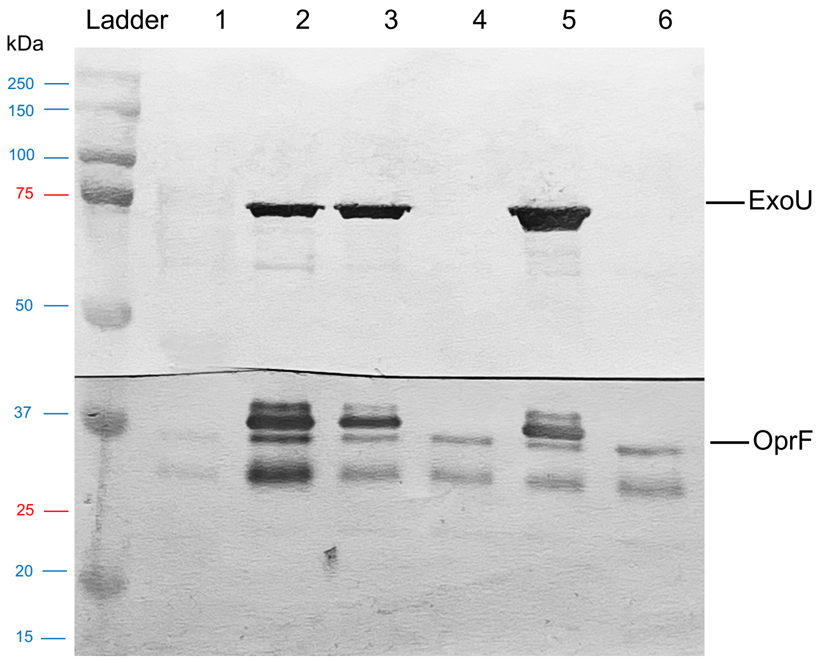

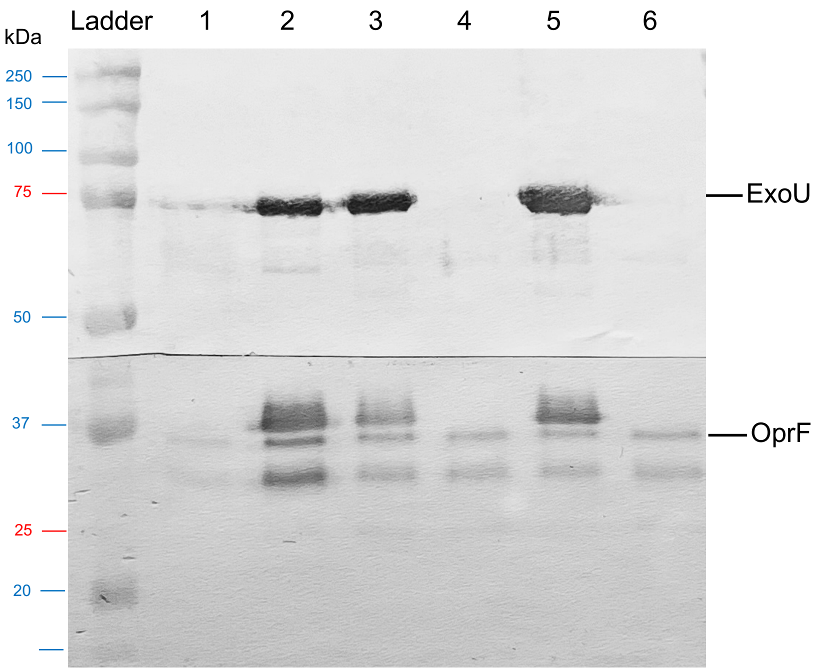

Pellet (S1 Fig)

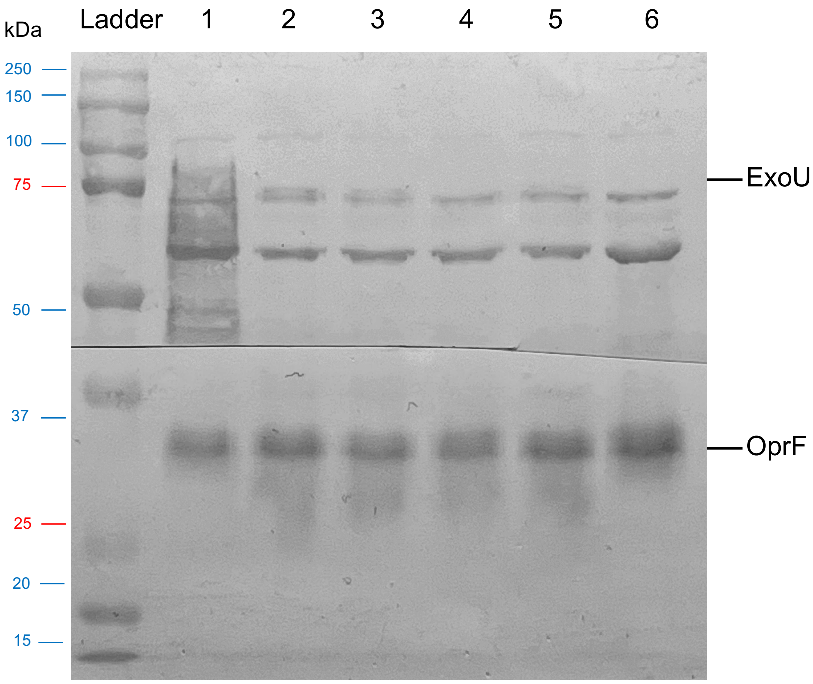

Well	Name
1	ATCC9027
2	PA14
3	ID4365
4	IDrsmA
5	IDrsmA + pUCrsmA
6	IDrsmA + pUCP20

Biological replicates:

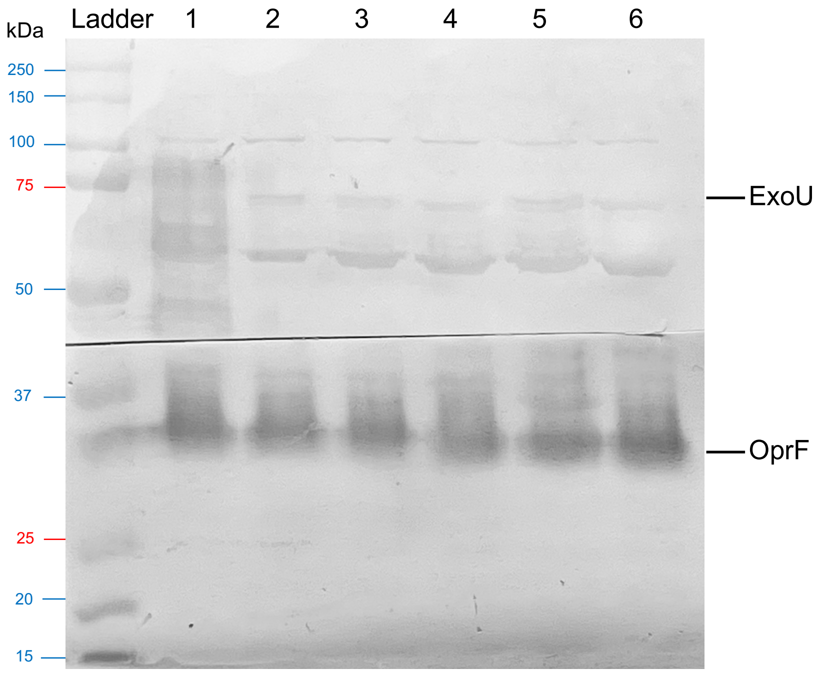

Well	Name
1	ATCC9027
2	PA14
3	ID4365
4	IDrsmA
5	IDrsmA + pUCrsmA
6	IDrsmA + pUCP20

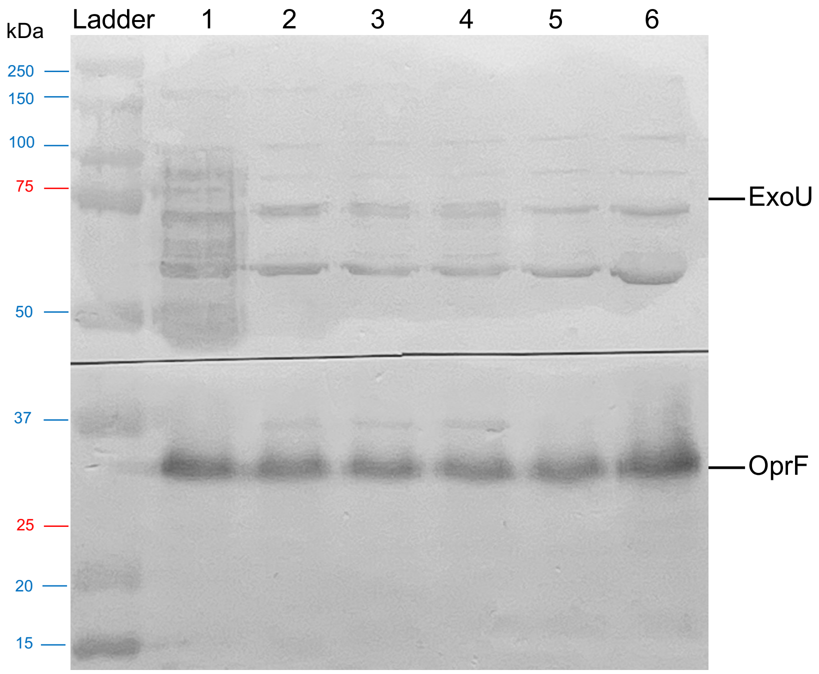

Supplement: S1 Raw Images — (PDF) [file pone.0337097.s007.pdf]
