# Supplementary material for: The rsmA mutant from Pseudomonas aeruginosa ID4365 is a non-virulent strain that is suitable for pyocyanin and phenazine-1-carboxylic acid production
Source: PLoS One. 2025 Dec 4;20(12):e0337097. doi: 10.1371/journal.pone.0337097 (PMC12677446; doi:10.1371/journal.pone.0337097)
Supplement: Attachment — Submitted filename: Response to Reviewers.docx [file pone.0337097.s010.docx]

Reviewer #1: This manuscript presents evidence that an RsmA-deficiency mutant ID4365 of *Pseudomonas aeruginosa* is suitable for producing high amount of pyocyanin. The experiments provided are necessary and the results are reasonable and logical. English is very perfect. Only on problem should be clarified: pyocyanin is reported as one of virulent factors in many published works. The mutant *P. aeruginosa* IDrsmA produces much more pyocyanin in some medium and under specific optimal culture conditions.

Did you check whether the pyocyanin serves as virulence factor biosynthesized and released in other cells or animals (not *Galleria mellonella* and mice). This problem should be clarified and discussed in discussion section at least.

Thank you very much for your comment. We did not check it. However, considering the characteristics of the IDrsmA strain related to its virulence factors (such as lower levels of elastase and rhamnolipids, along with an inactivated T3SS), similar results could be obtained using additional animal or cell models. This is discussed in the discussion section, lines 477-482.

Reviewer #2: Dear author,

this is a good article that describes the production of substances of biotechnological interest in an avirulent strain, thereby simplifying the safety processes involved in obtaining these compounds. The manuscript is very well written and is relevant in the scientific field.

Thank you very much.
